# Germ granule-mediated mRNA storage and translational control

**DOI:** 10.1080/15476286.2025.2462276

**Published:** 2025-02-03

**Authors:** Hoang-Anh Pham-Bui, Mihye Lee

**Affiliations:** a Soonchunhyang Institute of Medi-Bio Science, Soonchunhyang University, Cheonan-si, Korea; b Department of Integrated Biomedical Science, Soonchunhyang University, Cheonan-si, Korea

**Keywords:** RNA granule, germ cells, post-transcriptional regulation, mRNA storage, translational control, phase separation

## Abstract

Germ cells depend on specialized post-transcriptional regulation for proper development and function, much of which is mediated by dynamic RNA granules. These membrane-less organelles form through the condensation of RNA and proteins, governed by multivalent biomolecular interactions. RNA granules compartmentalize cellular components, selectively enriching specific factors and modulating biochemical reactions. Over recent decades, various types of RNA granules have been identified in germ cells across species, with extensive studies uncovering their molecular roles and developmental significance. This review explores the mRNA regulatory mechanisms mediated by RNA granules in germ cells. We discuss the distinct spatial organization of specific granule components and the variations in material states of germ granules, which contribute to the regulation of mRNA storage and translation. Additionally, we highlight emerging research on how changes in these material states, during developmental stages, reflect the dynamic nature of germ granules and their critical role in development.

## Introduction

Oogenesis and early embryogenesis primarily depend on the post-transcriptional regulation of mRNA rather than on transcriptional control. This regulatory framework involves diverse molecular mechanisms that influence mRNA stability, localization, and translation, all of which are crucial for developmental processes [1,2]. Newly synthesized mRNAs are processed in the nucleus and then transported to the cytoplasm [3]. The mRNAs designated for immediate protein synthesis are translated, however, another group of mRNAs is preserved in a dormant state [4] and is activated upon contact with specific cellular niches that require localized protein expression or in response to developmental or environmental signals [5,6]. Additionally, defective or unnecessary mRNAs are targeted for degradation [6]. These mRNA-regulatory mechanisms are orchestrated by a dynamic ensemble of RNA-binding proteins (RBPs) that often function within large ribonucleoprotein (RNP) complexes.

In a wide range of cell types across multiple species, RNA granules – membrane-less cytoplasmic structures enriched with RNAs and RNA-binding proteins – are essential for post-transcriptional regulations [7,8]. The key types of RNA granules include processing bodies (P-bodies) and stress granules, which are commonly found in many cell types, along with germline-specific granules such as polar granules, Balbiani bodies, sponge bodies and P granules. In recent decades, various types of germ granules have been identified, with their molecular functions and developmental significance extensively investigated [9,10]. In various invertebrate species, germ granules are observed in germ cells throughout all stages of development, whereas in mammals, they are primarily detected during the later stages of germ cell differentiation [11–13]. This suggests that the physiological roles of germ granules differ between species and can be more specialized at distinct stages of development. Despite these differences, germ granules display notable similarities in molecular composition and structure, indicating shared functions and potentially specialized roles.

Germ granules have diverse functions in germ cells, including RNA localization, storage, translational control, mRNA degradation, small RNA inheritance, small nuclear ribonucleoprotein (snRNP) assembly, and mitochondrial inheritance, which are essential for germline development [10,14–21]. Extensive studies have elucidated the molecular mechanisms and developmental roles of the germ granule-mediated regulation of maternal mRNA, underscoring the function of RNP granules as central hubs of post-transcriptional regulation. Recent technological advances have allowed the comprehensive characterization of germ granules, shedding light on the unexpected level of functional complexity arising from their heterogeneous composition and dynamic organization.

This review introduced representative RNA granules in the germ cells of various organisms (Table 1). We focused on post-transcriptional regulation mediated by germ granules and highlighted the recent progress in our understanding of spatially specified translational control and material state-mediated mRNA regulation within the granule.

**Table 1.** Diversity of germ granules across species.

Granule types	Species	Suggested functions	References
Polar granules	*Drosophila*	Sequestering factors for the formation of embryonic germ cells	[36]
		Oocyte polarity	[36]
Sponge bodies or P -bodies	*Drosophila*	RNA storage, translational regulation, and localization	[48,134]
		Protecting oocytes from environmental stress conditions	[49]
Balbiani bodies	*Drosophila*	Mitochondrial inheritance	[38,50]
		Transport and/or localization of the germline specific RNAs to the oocyte posterior pole	[11,50]
Nuage	*Drosophila*	piRNA processing involved in the protection of genome in the germline cells	[14]
P granules	*C. elegans*	mRNA processing	[58,59]
		Protecting germline transcripts from piRNA-mediated silencing	[61]
		Regulation of piRNA, miRNA and endo-siRNA	[60]
		Proper allocation of germline-specific RNAs and proteins to the developing embryo	[55]
P-body like granules or P-bodies	*C. elegans*	Storage sites for translationally-repressed maternal mRNAs	[58,58,62,63,65]
		Small RNA homeostasis and transgenerational silencing	[64]
		Maintaining the quality of oocytes by regulating RNA metabolism	[67]
Balbiani bodies	*Xenopus*	Maintaining a low activity of mitochondria and maternal RNAs	[136]
		Transport of components aiding germplasm assembly and germ cell specification	[73–75]
L-bodies	*Xenopus*	Proper embryonic patterning through RNA localization	[77]
Balbiani bodies	Zebrafish	Establishing the animal-vegetal axis and transfer RNAs and proteins to the vegetal pole	[79,80]
Perinuclear germ granules	Zebrafish	Maintaining germ cell totipotency/Germ cell specification and determination	[15,123]
		Post-transcriptional regulation	[123]
		Chromatin remodelling	[16]
Germ plasm	Avian species	Specification of PGCs	[17]
MARDO	Mammals	Storage and regulation of maternal mRNA in oocytes	[90]
Balbiani bodies	Mammals	Posttranscriptional RNA regulation and organelle distribution	[89,90]
		Oocyte survival	[88]
		Selection of healthy mitochondria during early oogenesis	[87]
		Protein and mRNA sequestration and retroposon inactivation	[11,76,84]
Intermitochondrial cement (IMC)	Mammals	Pachytene piRNA primary processing	[18]
		Mitochondria fusion	[19]
Chromatoid body (CB)	Mammals	Small RNA-mediated gene regulation	[20]
		Involvement in nonsense-mediated mRNA decay	[21]

## Characterization of RNA granule

Germ granules are membrane-less RNP assemblies found exclusively in the germline and are formed as biomolecule condensates containing proteins and RNAs without fixed stoichiometry. These granules are rich in key RNAs and proteins essential for the determination and maintenance of germ cells, and defective germ granule function results in developmental abnormalities. Following the observation that P granules exhibit characteristics akin to those of liquid droplets in *C.elegans*, phase separation has been suggested as a central mechanism driving germ granule formation [22]. It relies on proteins containing intrinsically disordered regions (IDRs) and low-complexity sequences (LCs). Those proteins form the core of various RNA granules through non-specific protein-protein interactions [23,24]. The formation of condensates also depends on RNA-binding domains and specialized protein-protein interaction domains of protein constituents [23,25,26]. Multivalent interactions facilitate the formation of complexes from numerous interacting partners, which further drive granule condensation [27,28]. Additionally, RNA-RNA interactions are critical for granule formation, depending on the RNA concentration. The granule condensation is further modulated by post-translational modifications of proteins [26,29].

Membrane-less granules with liquid-like properties enhance biochemical reactions by concentrating biomolecules in confined spaces. Additionally, they can also inactivate reactions by inhibiting the recruitment of other components into dense phases [30]. Liquid-like granules allow their components to remain mobile, facilitating dynamic exchanges with the environment [29,31,32]. These features are thought to characterize liquid-like germ granules and may contribute to their potential functions. However, some germ granules form non-liquid scaffolds by adopting a gel-like state from liquid-like state, in which concentrated proteins become kinetically arrested. These granules can protect biomolecules and further repress their activities, as observed in mature oocytes, where mRNAs are stored and transported to their destination in a translationally repressed state.

## Diversity of germ granules

### Germ granules in *Drosophila*

#### Germ plasm and polar granules

The germ plasm is a specialized cytoplasm located at the posterior region in mature oocytes and early embryos. In *Drosophila*, germ granules, known as polar granules, arise from the germ plasm and are enriched with maternal factors, including *nos* and *pgc* mRNAs, crucial for germ cell specification [33–35]. These granules emerge at the posterior pole of the developing oocyte, are passed on to embryos, and are ultimately incorporated into embryonic pole cells [36]. Nos proteins maintain a germ cell-specific cell cycle programme by repressing factors that promote somatic development [37], while Pgc proteins are essential for transcriptional silencing in primordial germ cells (PGCs) [34,35]. The germ plasm is also enriched in mitochondria, which expands the mitochondrial pool and facilitates the selective inheritance of healthy mitochondria [38]. Additionally, germ granules regulate the transposable elements (TEs) during germline development. Maternally deposited Piwi-interacting RNAs (piRNAs) in the germ plasm provide immunity against TEs by repressing TE transcription through the heterochromatin formation at TE loci [39,40]. Besides their functions in TE suppression, piRNAs also regulate mRNA in the germ plasm, in association with Aub and Tud proteins [41,42].

#### Sponge bodies or P-bodies

Sponge bodies in *Drosophila* oocytes and nurse cells are electron-dense, membrane-less structures in which the ribosome-free endoplasmic reticulum (ER) is embedded [72,73]. Originating from perinuclear nuage fragments, sponge bodies intermingle with P-body-like granules within the cytoplasm and contain components typically found in P-bodies [74]. Due to similarities in their components and functions, they are often grouped with P-bodies in the literature [75]. Sponge bodies are likely to transport materials between nurse cells and to the posterior ooplasm and undergo dynamic compositional changes [72,74,76]. They play a role in post-transcriptional regulation including RNA processing, localization, storage and translational regulation [43]. They may also protect oocytes from environmental stress by altering their morphology in response to varying conditions [43,45].

#### Balbiani bodies

In *Drosophila*, the Balbiani body (Bb) is a large and diffuse structure composed of RNAs, proteins, and various subcellular organelles, including mitochondria, ER, and Golgi [46,77]. The Bb, which initially appears near the nucleus, spreads throughout the cytoplasm and ultimately concentrates in the posterior oocyte cortex, where it integrates with other granules that develop from the germ plasm [13,46]. The Bb play a critical role in mitochondrial inheritance and is implicated in the localization of certain mRNAs, including *osk* RNA, which is crucial for germ plasm development. Bb-associated *osk* mRNAs indicate that Bb may be related to the recruitment of germline-specific RNAs to the posterior pole [11,46]. Given common components in Bb and sponge bodies, Bb may perform a similar function with sponge bodies [72].

### Germ granules in *Caenorhabditis elegans*

#### P granules

Germ granules in *C. elegans* were originally referred to as P granules, which was derived from P-lineage cells where they were first identified [78,79]. P granules is one of five types of perinuclear nuage compartments (P granules, Z granules, SIMR foci, Mutator foci, and E granules), which permits germ cells to produce diverse forms of small RNAs, increasing the diversity and regulation related to small RNA pathways in the germline [80]. During germ cell development, P granules are positioned near the nuclear membrane, but as oocyte maturation progresses, they become dispersed in the cytoplasm [78]. Their primary function is to designate germline cells by the accumulation of critical RNAs and proteins for development [51]. P granule components are implicated in multiple processes related to germ cell development, including proliferation and survival [81,82]. P granules participate in mRNA processing, storage and degradation, while also regulating miRNA and endo-siRNA pathways [47,48,50]. An additional function of P granules suppresses transposon activities through the piRNA pathway and also safeguard germline mRNA from piRNA-mediated silencing [49].

#### P-body like granules or P-bodies

Several types of P-body-like granules, characterized by P-body factors such as CGH-1 (DEAD-box RNA helicase) and CAR-1 (Sm-like protein), have been reported in oocytes and embryos of *C. elegans* [47,52,53]. These granules localize at the perinuclear P granules on the cytoplasmic side and interact with P granules in specific spatial arrangements during oogenesis [54,56]. Large P-body-like oocyte granules in *C. elegans* form under specific conditions (e.g. feminized or aged worms depleted of sperm), while P-bodies are smaller and dynamic, with an unclear biological function in young adult hermaphrodites [83]. In arrested oocytes, distinct large RNP condensates containing typical P-body factors and translational repressors act as repositories for translationally repressed maternal mRNAs [57]. The loss of CGH-1 disrupts the normal granule shape, leading to the destabilization of maternal mRNAs and translational activation, which impairs oocyte development [47,52,54,55].

Disassembly of large oocyte granules is triggered by sperm signals, which resumes meiosis and initiates the formation of embryonic P-body-like granules with different constituents and material phases [83,84]. In embryonic germline blastomeres, where P granules are preferentially segregated from the somatic blastomere through asymmetric division during embryonic cleavage, P-body-like granules interact with P granules, but they do not merge and retain their individual identities [53]. In embryonic somatic blastomeres, P-body-like granules recruit decapping activators, including Patr-1 (a homolog of Pat1), adopting a role in maternal mRNA degradation that differs from that of germline P-body-like granules.

### Germ granules in vertebrates

#### Balbiani bodies (Bb) in *Xenopus*

In *Xenopus*, the Bb is a large membrane-less condensate, also referred to as the mitochondrial cloud, which contains RNAs, proteins, and subcellular organelles [85,86]. The amyloid-like aggregate structure of Bb helps store and localize maternal mRNAs and other components, which are critical for establishing animal – vegetal polarity [11,86]. In oocytes, the Bb carries a specific set of translationally silent mRNAs, including those necessary for germ cell formation and embryonic patterning such as *dazl*, *syntabulin*, and *grip2a* mRNAs [59,87]. During oocyte maturation, the Bb moves towards the vegetal pole and transports mRNAs, mitochondria, and other germ plasm components to the vegetal cortex, where it participates in axial patterning and germ plasm specification [59–61]. The Bb subsequently disintegrates in later oogenesis stages and disappears completely during oocyte maturation, with its contents remaining anchored to the vegetal cortex [11,61,70].

#### Localization-bodies (L-bodies)

L-bodies are large cytoplasmic RNPs in *Xenopus* oocytes, found at the vegetal pole, particularly prominent during stages II and III of oogenesis [62]. These biomolecular condensates have an irregular shape and consist of relatively non-dynamic RNA components alongside more dynamic protein components [62]. L-bodies contribute to the localization of maternal RNAs that are critical for embryonic patterning [62,88].

#### Balbiani bodies (Bb) in zebrafish

Similar to *Xenopus*, the Bb directs the localization of maternal RNAs, proteins, electron-dense germ plasm, and subcellular organelles to the vegetal cortex during oogenesis [11,61,63,64]. These RNP condensates consist of homotypic clusters of specific RNAs, including *dazl*, *dnd*, *nos-3*, *rgs14a*, and *vasa/ddx4*, which are essential for distinguishing germline cells from somatic lineages during early embryonic development [89,90].

#### Balbiani bodies (Bb) in mammals

In mammals, Bb contains RNAs, proteins, Golgi, ER, and mitochondria, but lacks electron-dense granulofibrillar structures observed in *Xenopus* and zebrafish [71,85,91,92]. The characterization of the Bb in mice remains controversial, particularly due to the report describing an inconsistent presence of mitochondrial clusters surrounding the spherical Golgi structures [93]. Unlike in other species, where the Bb relocates to the posterior cortex, the mammalian Bb disperses uniformly within the cytoplasm [92], which supports the hypothesis that mammalian embryonic patterning is not dependent on localized maternal determinants. However, mammalian Bb may still contribute to post-transcriptional regulation and organelle distribution, as observed in other organisms [11,67–69,86].

#### Mitochondria-associated membrane-less compartments (MARDO) in mammals

In mammalian oocytes, mRNAs are sequestered in a translationally silent state in mitochondria-associated membrane-less compartments (MARDOs). During oocyte growth, the assembly of MARDOs is driven by an increased mitochondrial membrane potential. MARDOs become more evident in fully grown oocytes, coinciding with peak of mitochondrial activity and dissolve during the transition from meiosis I to meiosis II [66]. At the premeiotic and meiosis I stages, P-body proteins are found in MARDOs, where translationally repressed mRNAs are stored and protected against degradation [66,94]. Some of these mRNAs are translationally activated during oocyte maturation or fertilization [66]. Although both MARDO and Bb include mRNAs and mitochondria, they emerge at distinct stages of oogenesis and have different composition and physical characteristics [66,95].

## Regulation of mRNAs in germ granules

### mRNA localization and translational repression in *Drosophila* sponge bodies

In *Drosophila*, several maternal mRNAs, including *bcd*, *osk, nos*, and *grk*, which are essential for anterior-posterior embryonic pattern specification, are transported from nurse cells to oocytes during which they remain translationally repressed [96–98]. Sponge bodies are characterized by the enrichment of several key proteins, which are implicated in the control of these localized mRNAs, supporting the involvement of sponge bodies in mRNA storage and trafficking. Exu proteins are associated with *bcd* mRNAs and are essential for its anterior localization. Exu-containing RNP assemblies, presumably sponge bodies, move similarly to that of *bcd* mRNA in oocytes [99,100]. The protein constituents of sponge bodies, such as Bru, Cup, Orb, and Hrp48, are involved in transporting *osk* mRNA to the posterior pole, where the pole plasm contains large RNA complexes known as polar granules or germ granules [76,101–105]. Before their localization at the pole plasm, *osk* mRNAs are translationally repressed within sponge bodies, which is mediated by the sequence-specific translation repressor Bru [106,107]. Bru interacts with Bruno response elements (BREs) in the 3’ untranslated region (UTR) of *osk* mRNA via three RNA recognition motifs (RRMs), forming large RNP complexes [108]. Bru recruits an eIF4E-binding protein, Cup that interferes with the interaction between eIF4E and eIF4G, thereby preventing ribosomal recruitment and translation initiation [101].

### mRNA storage and translational repression in *C.* *elegans* P-Body like granules

In *C. elegans*, P-body-like granules are involved in mRNA storage and translational repression. During oocyte arrest, mRNAs that are translationally repressed, along with their associated RNA-binding regulators, are recruited to P-body-related particles. The 3′ UTR elements responsible for translational repression play a role in the localization of these mRNAs to the granules [54]. P-body factors, such as CAR-1, are critical for the recruitment of repressed mRNA to granules and the formation or stabilization of these granules [54]. Another P-body factor, CGH-1, a DEAD-box RNA helicase, is implicated in stabilizing hundreds of mRNAs in germ cells, while CGH-1 is known to promote mRNA decay in P-bodies of somatic cells [47]. In arrested oocytes, P-body droplets exhibit semi-liquid properties that initiate the repression of RNPs, preserving them for long-term storage until oogenesis resumes [84]. CGH-1 is crucial in regulating the supramolecular states of specific RNP components to prevent the transition to non-dynamic solid phases.

### Selective translational activation in *Drosophila* polar granules

The confinement of Nos proteins to the posterior pole of *Drosophila* embryos is ensured by the selective translation of *nos* mRNA at the specific region [109,110]. Polar granules in the posterior region serve as active translation sites for *nos* mRNAs. In contrast, unlocalized *nos* mRNAs are translationally repressed by the RNA-binding protein Smg. Smg bound to the *nos* 3′ UTR recruits Cup along with the CCR4-NOT deadenylation complex, which induces the deadenylation and translational repression of *nos* mRNA [109,111–113]. This repression is relieved by the inactivation of Smg through the interaction between Smg and Osk, which is facilitated by a localization complex comprising Osk, Tud, and Vas proteins in polar granules [114,115].

The post-transcriptional regulation of *nos* mRNA is also influenced by the PIWI protein Aub, which occurs at the periphery of germ granules [116,117]. piRNAs and Piwi proteins primarily function to repress transposons, however, they also regulate maternal mRNAs in germ cells. In *Drosophila*, maternally transferred piRNAs bound to Aub target mRNAs via incomplete base pairing, destabilizing maternal mRNAs in the soma but stabilizing them in the pole plasm [41,116,118]. Interestingly, Aub interacts with eIF4E, PABP, and subunits of the eIF3 complex, which are critical for promoting all of the steps of translational initiation [117,119]. This finding suggests that Aub also contributes to the translational activation of *nos* mRNAs in germ granules. The function of Piwi in translational control is evolutionary conservation. MIWI and piRNAs participate in translational activation through the association with PABP and several eIF3 subunits during mouse spermatogenesis [120].

Polar granules form within the germ plasm during late oogenesis and are anchored at the posterior cortex. As described above, polar granules protect maternal mRNAs and prevent their translational repression, thus facilitating posterior region-specific protein enrichment in early embryos [36]. Maternal mRNAs sequestered within the granule structure are stabilized, although extensive degradation of maternal mRNAs occurs before the zygotic transcriptional activation to establish the maternal-to-zygotic transition [5,121]. However, germ granules increase in size through fusion and recruit decapping protein, Dcp1, after pole cell formation, leading to a functional shift [122,123]. Enlarged germ granules selectively degrade specific mRNAs (e.g. *nos* and *pgc*), while leaving others (e.g. *CycB*) intact throughout embryonic development. The decapping activators Edc3 and Patr-1 facilitate the assembly of decapping complexes to the germ granules. Silencing of *edc3* and *patr-1* reduces granule size and impairs the recruitment of DCP1, which stabilizes *nos* and *pgc* mRNAs in germ granules.

## Spatial control of mRNA translation within germ granules

### Translational activation in the periphery of zebrafish perinuclear germ granules

**Figure 1.**
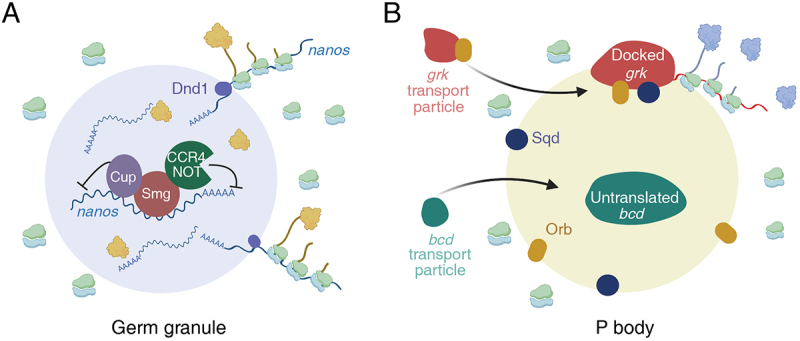
Translational activation in the periphery. (A) the accumulation of Dnd1 (dark purple) and *nanos3* mRNA at the periphery of perinuclear germ granules is essential for translating *nanos3* mRNAs in zebrafish. The repression of translation at the core of the granule involves Smg (red), a cytosolic RNA-binding protein that binds to the 3′ UTR of *nanos3*, recruiting the translational repressors Cup (purple) and the CCR4-NOT deadenylation complex (green). (B) In *Drosophila*, *grk* mRNA forms dynamic RNP particles and is translated at the outer edge of P bodies, where the translational activator Orb (yellow) and the anchoring factor Sqd (dark blue) are concentrated. In contrast, *bcd* mRNA translation is suppressed as it localizes to the core of P bodies, a region lacking ribosomes.

Abbreviations: *bcd*, *bicoid;* CCR4-NOT, Carbon Catabolite Repression – Negative On TATA-less; Dnd1, Dead end 1; eIF4E, Eukaryotic Translation Initiation Factor 4E; *grk*, *gurken*; Orb, oo18 RNA binding; Sqd, Squid; Smg, Smaug; UTR, untranslated region. Created with BioRender.com.

Germ granules contain mRNAs that encode proteins critical for germline development with their translation tightly regulated. These structures protect RNAs from degradation and store them in a dormant state, thereby maintaining an RNA reservoir for developmental stage-specific translation [124,125]. Thus, initiating translation requires interactions of the transcripts with translation machinery outside the granules. In zebrafish, the spatial control of the transcript *nanos3* mRNA, localized in the germ granule, was recently found to facilitate the temporal regulation of translation during development. Westerich et al. reported that *nanos3* mRNAs localize at the outer edge of the perinuclear germ granule through *in vivo* imaging throughout germline development (Figure 1A). Interestingly, at earlier developmental stages when *nanos3* translation is inhibited, the mRNA is predominantly concentrated in the core of the granule. This suggests that *nanos3* mRNA localization within the granule is linked to the regulation of its translation. The conserved RNA-binding protein Dnd1 is known to prevent miRNA-mediated translational suppression of *nanos3* mRNA, allowing Nanos protein synthesis. Westerich et al. also found that Dnd1 proteins are associated with *nanos3* mRNA and are essential for maintaining *nanos3* mRNA clusters at the outer edge of germ granules, where active translation can occur. Although translation initiation factors are spread throughout the granules, ribosomes are distinctly enriched in the peripheral region of the granules where *nanos3* mRNA clusters overlap, indicating position-specific translational activity. This was further supported by the accumulation of polysomes and contact of the ER with the circumference of the granules. The analysis of green fluorescent protein (GFP) reporters containing *nanos3* 3’UTR suggested that Dnd1-mediated regulation of *nanos3* mRNA relies on the 3’UTR element [65]. Translational activity also influences the localization of *nanos3* mRNA to the periphery. Given the functional importance of Nanos3 proteins, germ granule structure-dependent translational regulation constitutes the critical developmental programme for gene expression control.

### Spatially-regulated translation in *Drosophila* P-bodies

Similar spatial position-dependent translation control of *grk* and *bcd* mRNAs that encode key players in embryonic axis specification, was discovered in the P-bodies of *Drosophila* oocytes. During mid-oogenesis, *grk* mRNAs localize to the anterior-posterior corner of oocytes, closely associated with the nucleus, while *bcd* mRNAs are deposited in the anterior cortex. Both mRNAs are translationally repressed during transport through microtubules and dynein [126–128]. Anchored *grk* mRNAs form dynamic RNP particles at the periphery of P bodies, where the anchoring factor Sqd and the translational activator Orb are concentrated (Figure 1B). This localization triggers the translation of *grk* at the anterior, facilitating the transmission of a transforming growth factor-α (TGF-α) signal to the overlying follicle cells [129,130]. In contrast to the translational activation of *grk* mRNAs at the outer edge of P bodies, *bcd* mRNAs are subjected to translational repression as it enters the ribosome-depleted core of P bodies in oocytes (Figure 1B). Following egg activation, in embryos, *bcd* mRNA disassociates from the P bodies, becomes translationally active, and establishes an anteroposterior morphogenetic gradient [130,131]. In addition, excess *grk* mRNAs are sequestered inside P-bodies, where they undergo translational repression, similar to *bcd* mRNAs in oocytes [130]. These findings indicate that the positioning of mRNAs within granules is critical for differential translational control and underscore the need to investigate the underlying mechanisms that govern the partitioning of granules with different proteins and mRNAs in translational regulation.

### Spatial pattern of translational control in *Drosophila* germ granules

Recent cutting-edge imaging techniques have revealed translational dynamics at a single mRNA resolution [132,133]. As described above, polar granules play a key role in the temporal translational activation of maternal mRNAs through the Osk-mediated antagonization of Smg-dependent translational repression. Chen et al. visualized the spatiotemporal pattern of *nos* translation using the Suntag method to monitor translation at the single-molecule level. Their analysis revealed that the translation of *nos* primarily occurred at the surface of the polar granule. Translating polysomes were observed around the surface of polar granules and the the 3′ UTR of translated *nos* mRNAs was buried within the granule. In addition, Osk regulates the sequestration of the translation repressor Smg. These results highlight the molecular mechanisms based on the spatial distribution of mRNAs and proteins within granules.

## Regulation of mRNA via the material state dynamics of granules

### Phase transition of P-body in oocyte-to-embryo transition in *Drosophila*

**Figure 2.**
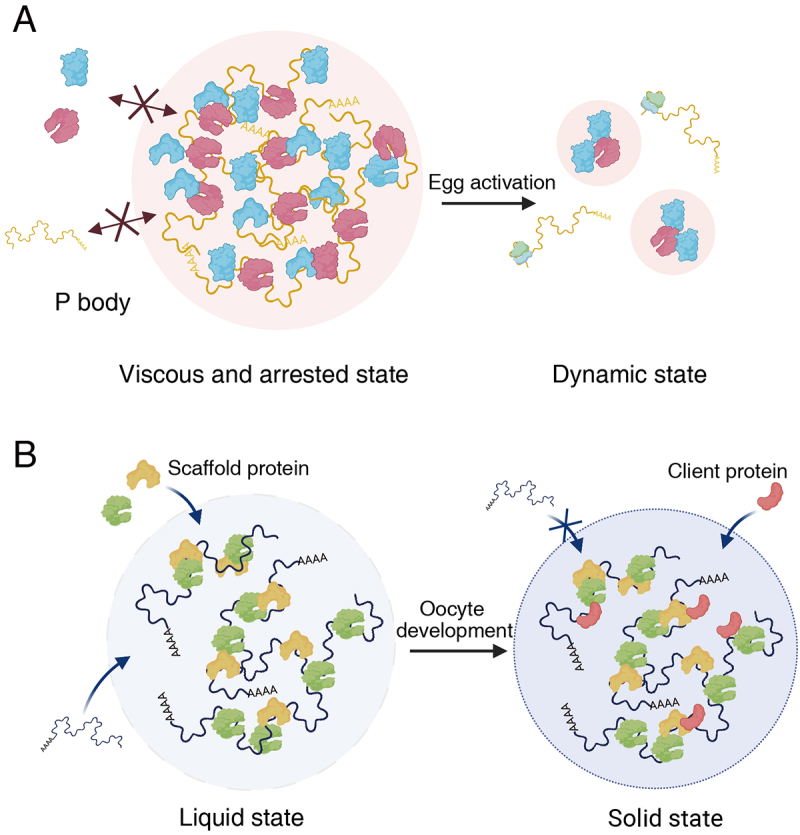
mRNA regulation via the material state dynamics. (A) P body condensates are in an arrested physical state that limits protein exchange between the P body and the cytoplasm, facilitating the storage of mRNA in *Drosophila* mature oocytes. Upon egg activation, the material properties of P bodies change, allowing the release of stored mRNA for translation. (B) The granules containing *osk* mRNA (presumably sponge bodies) exhibit solid-like properties in *Drosophila* oocytes. The integration of mRNA with scaffold granule proteins leads to the formation of amorphous, spherical, and dynamic condensates, which rapidly transition into a solid state that selectively enriches client proteins. Created with BioRender.com.

RNA granules show a spectrum of fluidity, ranging from liquid-like to gel-like and solid-like forms, which can change progressively and reversibly to facilitate specific biological functions; however, the underlying mechanisms remain unclear [134,135]. In germ granules, material state transition facilitates differential translation control in oocytes and early embryos (Figure 2A) [44]. In *Drosophila* oocytes, the P body containing Me31B, which is pivotal for regulating key maternal factors, including *grk* and *bcd* mRNAs, exhibits an arrested physical state [44]. The dense and less dynamic features of P-body condensates are governed by electrostatic and hydrophobic interactions involving both extrinsic and intrinsic factors (e.g. RNA, actin, disordered proteins, multivalent interactions, and IDRs). This solid-like material may confer mechanical stability and generate a molecular mesh that filters molecules, thereby suppressing their activities and protecting biomolecules [86,136]. Consequently, this property of the P-body is crucial for the storage of *bcd* mRNA in mature oocytes [44]. Similarly, the solid-like material state of Bb supports the long-term storage of macromolecules during the dormant phase of vertebrate oocytes [58]. In addition, the translation of *grk* mRNA exclusively at the periphery of the P-body, as observed by Weil et al., may be attributed to the integrity of the P-body. The translation of *bcd* mRNA has been reported to be initiated only when the mRNA is exposed outside the P bodies in previous studies [130,137]. Upon egg activation, alterations in the properties of P-bodies lead to the release of *bcd* mRNA, allowing its translation to begin [44].

### Solid-like phase of osk-containing granule in *Drosophila*

In *Drosophila*, Osk proteins, the determinants that lead to the assembly of the pole plasm, are tightly regulated for their proper accumulation in the posterior region of oocytes for embryonic axis formation and germline cell specification. To generate spatial restriction of Osk proteins, *osk* mRNAs are transported posteriorly and translated after proper localization [106,138,139]. To regulate localization and translation, *osk* mRNAs associate with proteins, including Bru, Hrp48, and PTB, through multivalent interactions [140]. Bose et al. recently found that the RNP complex containing *osk* mRNA forms granules with solid-like properties, which are potentially sponge bodies, in oocytes (Figure 2B) [141]. RNP component proteins containing disordered regions and *osk* mRNA 3’UTR were reconstituted into the spherical, liquid-like condensates that rapidly undergo the phase transition into the solid state *in vitro*. The liquid phase is essential for RNA incorporation, while the solid phase contributes to the assembly of specific proteins. Manipulation of osk-containing granules from solid-like to liquid-like phase impairs localization and translation control, leading to abnormalities in embryonic axis formation. This study highlights the physiological role of the material state in granule-mediated mRNA regulation.

### Post-translational modification of germ granules in *Drosophila*

The post-translational modification of the constituent proteins in RNP condensates is known to affect phase separation and regulate the physical properties of germ granules [142,143]. Fajner et al. recently found that Hecw ubiquitinates Fmrp within germ granules, possibly sponge bodies. This modification increases the phase separation propensity of Fmrp and decreases its affinity for ribosomes or target mRNAs. Depletion of Hecw induces the transition of granules from a liquid- to a gel-like state and downregulates the translational repression activity of Fmrp on its target *orb* mRNA. Taken together, these results indicate that Hecw interacts with the component of the RNP complex and modulates its assembly during oogenesis, contributing to translational control [144–147].

## Conclusion remarks and perspectives

Germ granules are specialized cellular structures formed through the condensation of RNAs and proteins, and are regulated by multivalent and dynamic interactions between biomolecules. These granules enrich specific components and segregate them from cytoplasmic factors. Selective compartmentalization can enhance biochemical reactions by increasing local concentrations or suppress reactions by sequestering molecules from their interactors. Such dynamic regulation allows for the intricate and multilayered regulation of maternal mRNAs at the post-transcriptional level.

Over the past few decades, diverse types of germ granules have been discovered, with their features extensively documented in previous reviews [9,10]. Here, we focus on well-characterized mechanisms mediated by germ granules that exemplify their molecular roles in post-transcriptional regulation. Furthermore, we highlighted recent studies on the specific positioning of granule components within germ granules and the variations in their material states, which play a key role in the delicate balance and finely tuned control of mRNA storage and translation. These material states are dynamic, undergoing changes in response to various cellular conditions, thereby reflecting the adaptive nature of germ granule function.

Recent advances in the mechanistic understanding of germ granule assembly and its functions have emerged with cutting-edge techniques. Since germ granule proteins are diffusely present in the cytoplasm as soluble proteins, but are concentrated within granules, conventional approaches to studying the function of individual proteins through genetic ablation are inadequate for dissecting their role as granule components. Extensive *in vitro* assays have helped us characterize the mechanisms by which different biomolecules condense into granules and how their physical states are regulated. In addition, high-resolution imaging has revealed that RNA and protein biomolecules are non-homogenously distributed, and the differential material properties are organized within the granules. However, the extent to which these unique characteristics of the granules contribute to their biological functions remains incompletely understood and warrants further investigation.

## Data Availability

No new data were generated or analysed in this review.
